# Severe asthma: anti-IgE or anti-IL-5?

**DOI:** 10.3402/ecrj.v3.31813

**Published:** 2016-11-07

**Authors:** Evgenia Papathanassiou, Stelios Loukides, Petros Bakakos

**Affiliations:** 1 2nd Department of Respiratory Medicine, Attikon University Hospital, National and Kapodistrian University of Athens, Athens, Greece; 2 1st Department of Respiratory Medicine, ‘Sotiria’ Hospital of Chest Diseases, Medical School, National and Kapodistrian University of Athens, Athens, Greece

**Keywords:** severe asthma, monoclonal antibodies, IgE, IL-5, inflammation, asthma control

## Abstract

Severe asthma is a discrete clinical entity characterised by recurrent exacerbations, reduced quality of life and poor asthma control as ordinary treatment regimens remain inadequate. Difficulty in managing severe asthma derives partly from the multiple existing phenotypes and our inability to recognise them. Though the exact pathogenetic pathway of severe allergic asthma remains unclear, it is known that numerous inflammatory cells and cytokines are involved, and eosinophils represent a key inflammatory cell mediator. Anti-IgE (omalizumab) and anti-IL-5 (mepolizumab) antibodies are biological agents that interfere in different steps of the Th2 inflammatory cascade and are licensed in severe asthma. Both exhibit a favourable clinical outcome as they reduce exacerbation rate and improve asthma control and quality of life, while mepolizumab also induces an oral steroid sparing effect. Nevertheless, it is still questionable which agent is more suitable in the management of severe allergic asthma since no comparable studies have been conducted. Omalizumab's established effectiveness in clinical practice over a long period is complemented by a beneficial effect on airway remodelling process mediated mainly through its impact on eosinophils and other parameters strongly related to eosinophilic inflammation. However, it is possible that mepolizumab through nearly depleting eosinophils could have a similar effect on airway remodelling. Moreover, to date, markers indicative of the patient population responding to each treatment are unavailable although baseline eosinophils and exacerbation rate in the previous year demonstrate a predictive value regarding anti-IL-5 therapy effectiveness. On the other hand, a better therapeutic response for omalizumab has been observed when low forced expiratory volume in 1 sec, high-dose inhaled corticosteroids and increased IgE concentrations are present. Consequently, conclusions are not yet safe to be drawn based on existing knowledge, and additional research is necessary to unravel the remaining issues for the severe asthmatic population.

Asthma is a heterogeneous disease characterised by chronic airway inflammation. It is defined by a history of symptoms including wheeze, shortness of breath, chest tightness and cough that vary over time and in intensity, together with variable expiratory airflow limitation (1). Nowadays, inhaled corticosteroids (ICS) and bronchodilators constitute the mainstay of asthma treatment. Despite the multifaceted effectiveness of asthma pharmacotherapy in mild and moderate forms of the disease, patients with severe asthma often depend on oral steroids in order to adequately control their asthma. This group of patients is at increased risk of exacerbations that may require hospitalisation. They also have a reduced quality of life not merely due to the disease itself but also due to the side effects of the required treatment. According to the recent european respiratory society/american thoracic society (ERS/ATS) consensus, severe asthma is defined as asthma that requires treatment with high-dose ICS plus one more controller (and/or oral corticosteroids) in order to be controlled or remains uncontrolled despite the above treatment or becomes uncontrolled with the reduction of high-dose ICS or oral corticosteroids. Although this group accounts for only around 5% of asthmatics, it contributes to approximately 50% of the economic costs of asthma; therefore, the development of novel therapies is essential towards optimal treatment of severe asthma (2, 3). To date, two monoclonal antibodies, anti-IgE and anti-IL-5, are available but selection criteria between the two have not yet been established (4, 5). The aim of the present review is to provide an overview of the evidence behind the use of anti-IgE and anti-IL-5 in severe asthma and possibly to assess the clinician's judgment in choosing the right treatment for the right patient.

## Methods

We searched Medline with language restriction (only articles published in English). The following search terms were used: ‘asthma’, ‘severe asthma’, ‘anti-IL-5’, ‘anti–IgE’ and ‘omalizumab’. The studies selected were then further analysed for data extraction, including searching the reference lists. Particular attention was given to studies in severe asthma.

## Anti-IgE

### Introduction

It is estimated that more than 50% of people with poorly controlled asthma have allergic immunoglobulin E (IgE)-mediated asthma and, therefore, may benefit from treatments targeted at IgE. In fact, several biological agents that interfere either in the synthesis or in the signalling pathway of IgE have emerged or are under investigation. Omalizumab, the only biological anti-IgE agent currently licensed for use in humans, is a recombinant DNA-derived humanised IgG1 monoclonal antibody. It was originally constructed as a murine antibody selectively binding to human IgE (6).

### Mode of action-effect on inflammation

Omalizumab binds exclusively to circulating IgE in the blood and interstitial space and consequently promotes the depletion of circulating IgE. It also inhibits IgE binding to high-affinity (FcɛRI) or low-affinity receptors (FcɛRII) on basophils, mast cells and dendritic cells. Omalizumab cannot bind to IgE that is already bound to FcɛRI, thus avoiding the FcɛRI cross-linking that could potentially lead to anaphylaxis. Although it does not have a direct effect on FcɛRI levels, downregulation on cells bearing the receptor actually occurs, mediated through the depletion of free IgE (7–12). Omalizumab interferes with the inflammatory cascade by reducing serum IgE levels and FcɛRI receptor expression on key cells. This results in the inhibition of the release of inflammatory mediators from mast cells and diminished recruitment of inflammatory cells, especially eosinophils, into the airways (13–16).

Apart from the effect on blood and sputum eosinophil count, a decrease in markers of eosinophilic airway inflammation such as serum eosinophil cationic protein, endothelin-1(ET-1) in exhaled breath condensate and fraction of exhaled nitric oxide (FeNO) has also been observed (17, 18). It has been suggested that ET-1 plays an important role in the development of severe bronchial hyperreactivity and airway remodelling through enhancing bronchial smooth muscle cells’ proliferation and subepithelial fibrosis (19). Since omalizumab treatment affects the inflammatory process in multiple ways, it is possible that long-term therapy with omalizumab may have a beneficial effect on airway remodelling either by inhibiting its progress or by reversing changes already present. Indeed, omalizumab in murine models and *in vitro* experiments was capable of inducing a decrease in markers of remodelling, such as peribronchial collagen III/V deposition, hydroxyproline and α-smooth muscle actin (20). Moreover, omalizumab treatment in patients with severe persistent asthma was associated with a significant reduction in reticular basement membrane (RBM) thickness, bronchial smooth muscle proteins and other indices of airway wall thickness compared to standard care (21–23).

### Indications

Omalizumab was approved by the United States (Food and Drug Administration (FDA)) in 2003 and by the European Union (European Medicines Agency) in 2005 as an add-on treatment for patients aged >12 years with severe persistent allergic asthma. Serum total IgE levels should be in the range 30–700 IU/mL in the USA. In Europe, serum total IgE ranges are from >30 to <1,500 IU/mL in adults and children over 6 years old. The dose (mg) and dose frequency of omalizumab are based on the serum total IgE level (IU/mL) and the patient's body weight (kg). Based on a calculation, omalizumab is given by a subcutaneous injection every 2 or 4 weeks. Initial treatment response is evaluated at 16 weeks, and treatment is continued in patients showing a response at that time.

### Clinical outcomes

Nowadays, a large body of evidence from randomised controlled trials and real-life studies is available demonstrating that anti-IgE treatment reduces exacerbation rates and improves asthma control in patients with severe allergic asthma (Table 1).

**Table 1:** Anti IgE treatment in severe asthma and clinical outcomes

Study	No. of patients	No. of patients treated with anti-IgE	Outcome
Bousquet et al. (24)	4,308	2,511	⇓Exacerbation 38% ⇓Hospital admissions 52% ⇓ER visits 47%
Humbert et al. (25)	419	419	⇓Exacerbation rate 26% ⇑ QoL ⇑ Morning PEF ⇓Symptom scores
Normansell et al. (26)			⇓Exacerbation ⇓Hospitalisation ⇓ICS daily dose
Abraham et al. (27)			⇓Exacerbation ⇓Hospitalisations ⇓ER visits ⇓OCS 30–66%
Busse et al. (28)	419	208	⇓Number of days with asthma symptoms 24.5% ⇓Exacerbations ⇑ Asthma control: omalizumab+lower doses of inhaled glucocorticoids (*p* <0.001) and LABA (*p=*0.003)
Hanania et al. (29)	850	427	⇓Mean daily albuterol puffs ⇓(−0.27 puff/day) ⇓Exacerbations ⇑ AQLQ(S) scores ⇓Asthma symptom score
Chen. et al. (30)			⇓Total ICS dose ⇓SABA ⇓LTRA

ER, emergency room; PEF, peak expiratory flow; ICS, inhaled corticosteroids; LABA, long acting beta2 agonists; AQLQ, Asthma Quality of Life Questionnaire; SABA, short acting beta2 agonists; LTRA, leukotriene receptor antagonists.

Evidence from randomised trials is not only confirmed but also expanded from everyday clinical experience.

### Lung function

Omalizumab treatment improves lung function, yet modestly (26). In the INNOVATE trial, a moderate but at the same time statistically significant improvement in forced expiratory volume in 1 sec (FEV_1_) and morning peak expiratory flow (PEF) from baseline was observed compared to that in placebo group (25). Moreover, in a real-life setting, a significant improvement of FEV_1_ was observed after 4 years of omalizumab treatment (31).

### Asthma-related quality of life

Anti-IgE treatment promotes a better disease control, and this is verified by improvement in quality of life. Significantly greater improvement in the overall Asthma Quality of Life Questionnaire (AQLQ) occurred in omalizumab group, and this remained during both steroid stable and steroid reduction phases (26). In another study, an excellent rate on the physician-rated global evaluation of treatment effectiveness scale was achieved for 74.6 and 81.6% of patients at 16 weeks and 2 years, respectively. Interestingly, this pattern was maintained for patients with ‘off-label’ IgE >700 IU/mL (32).

### Safety and tolerability

As the numbers of patients in clinical trials increase and clinical use has expanded, more data on the safety of the short- and long-term use of omalizumab are available. An analysis of over 7,500 patients participating in clinical trials of omalizumab and of 57,300 patients included in post-marketing safety follow-up monitoring presents a more than adequate safety profile. The major adverse effect recorded is anaphylaxis. The incidence of anaphylaxis reported in clinical trials is 0.14% in omalizumab-treated patients, 0.07% in control patients in clinical trials and 0.2% with omalizumab treatment from post-marketing data (33). Adverse effects were generally of mild-to-moderate severity and of short duration. However, injection site reactions were more frequent in the omalizumab group (19.9% vs. 13.2%) (34). Based on previous observations from clinical trials, concern arose about the relationship between omalizumab treatment and malignancies’ development. A causal relationship seems unlikely as the overall incidence of observed malignancy is rare in omalizumab-treated patients and comparable to that in the general population (35–37). Recently, concerns about the cardiovascular and cerebrovascular safety of omalizumab have been raised by the United States FDA, based on preliminary data from the EXCELS study (38). A retrospective pharmacovigilance analysis used reports of arterial thrombotic events submitted to the US FDA's Adverse Event Reporting System between 2004 and 2011 to evaluate the association of omalizumab with arterial thrombotic events. Omalizumab-treated patients reported a higher than expected number of arterial thrombotic events (OR 2.75). The majority of the arterial thrombotic event reports concerned myocardial infarction and stroke. Despite the large sample sizes, risk estimates from this study were not statistically significant. Given the clinical importance and the impact of such adverse effects on public health, as well as the fact that the causal relationship between omalizumab and these results has not yet been investigated, it is crucial to conduct additional epidemiological studies to test this hypothesis. Meanwhile, FDA recently (9/2014) added information about this potential risk to the drug label, and it is suggested that prescribers should cautiously prescribe omalizumab to asthmatic patients who have risk factors that contribute to arteriothrombotic events (39).

There is a theoretical possibility that treatment with omalizumab could increase susceptibility to helminth infection. A non-statistically significant increase in infection incidence with omalizumab was observed in a 52-week, randomised, placebo-controlled study in adults and adolescents with allergic asthma or allergic rhinitis at high risk of intestinal helminth infection (40). It is suggested that patients at high risk should be warned, especially when travelling to areas where these infections are endemic.

### Recognising the responders

It is important to recognise that not all patients respond to omalizumab treatment and that therapy cessation in non-responders will protect them from unreasonable drug exposure. Several studies have tried to identify clinical or laboratory characteristics that predict a good therapeutic response to omalizumab. In a pooled analysis of two randomised placebo-controlled trials, factors indicative of more severe asthma (history of emergency treatment, low FEV_1_ and high-dose ICS) were predictive of a greater relative response to add-on omalizumab (24, 41). It is noteworthy that in one study the baseline total IgE could be a predictor of response (42).

A potential arises through the observation that specific cut-off values for Th2 biomarkers (blood eosinophils, serum periostin and FeNO) could serve as predictors for omalizumab response (43).

### Duration of treatment

To date, guidelines concerning omalizumab's treatment duration do not exist. A clinical observation in a group of 18 patients, in which omalizumab treatment was stopped after 6 years of therapy, reported at 3-year follow-up improved or stable asthma control combined with lack of necessity for concomitant medication step-up. The reactivity of blood basophils to the leading perennial allergens (cat dander and house dust mites) remained low, at levels below those before the start of anti-IgE treatment (44, 45).

Overall, omalizumab therapy is associated with a distinct therapeutic impact on the full burden of severe asthma. Exacerbation rate, health care resource utilisation, symptom frequency and quality of life are positively affected. Possible effect on airway remodelling opens a new horizon in asthma therapy although some aspects still remain unclear regarding patients’ characteristics who will mostly benefit, optimal duration of treatment and long-term safety issues.

## Anti-IL-5

### Introduction

Eosinophils are a key inflammatory cell mediator in the pathogenesis of asthma (46). Asthma is usually characterised by eosinophilic airway inflammation and structural changes in the airway wall termed ‘remodelling’ (47). Remodelling may be the consequence of excessive repair processes following repeated airway injury, and there is increasing evidence that eosinophils may also be important in the pathophysiology of remodelling. In severe asthma, it has been shown that thickening of the subepithelial basement membrane was associated with increases in bronchial mucosal eosinophils (48). Eosinophil is the source of several molecules involved in remodelling processes such as TGF-α, TGF-β, VEGF, matrix metalloperoxidase-9, tissue inhibitor of metalloproteinase-1 and IL-13. Interleukin-5 (IL-5) is essential for eosinophil terminal differentiation, maturation and migration into the circulation and prolongs the survival of the cell in tissues (49). Other cytokines and chemokines also play a role in these processes (50, 51). The importance of IL-5 in asthma pathobiology has been elucidated by studies on anti-IL-5. These studies provided evidence not only for the possible role of anti-IL-5 as a therapy for asthma but also in clarifying the role of airway eosinophils in its pathobiology. Anti-IL-5 is a humanised monoclonal antibody that inhibits the bioactivity of IL-5. It blocks the binding of IL-5 to the alpha chain of the IL-5 receptor complex on the eosinophil cell surface. This inhibits IL-5 signalling and reduces the growth, differentiation, recruitment, activation and survival of eosinophils (52, 53).

### Early studies

The initial studies on IL-5 provided disappointing results. Two studies evaluating mepolizumab (monoclonal antibody against IL-5) in asthmatics (54, 55) demonstrated that the antibody effectively prevented the rise in blood eosinophils but did not affect clinical outcomes. Thus, it was concluded that IL-5 was important in the mobilisation and trafficking of eosinophils into the airways, but questioned the role of eosinophils in asthmatic responses. However, these conclusions were limited by issues raised about the study design, such as sample size and methodology (56). A larger study evaluated the efficacy of mepolizumab at two different doses (250 and 750 mg) on various asthma outcomes in a group of more than 300 patients with moderate to severe asthma with poor asthma control. In keeping with the other studies, the antibody caused an impressive reduction in blood eosinophils, but did not demonstrate any significant improvement in any of the clinical outcomes measured (57). In the same study, mepolizumab had a significant effect in reducing sputum eosinophil numbers, but in the low-dose treatment group this effect was only partial. The lack of ability of mepolizumab to completely abolish airway eosinophils, while having marked effects in reducing blood eosinophils, has previously been documented (58). These results, therefore, were interpreted as the ‘final nail in the coffin’ for anti-IL-5 as a treatment strategy for asthma.

However, there are two very important issues when evaluating the results of the initial anti-IL-5 studies that yielded disappointing results on asthma outcomes. The first is the study population, and the second is the primary outcome. The ideal study population for the evaluation of anti-IL-5 treatment would be a population of asthmatic patients with poorly controlled asthma, with high numbers of airway eosinophils and who are already under treatment with ICS.

The choice of the primary outcome in anti-IL-5 studies that target eosinophils is equally important. The study by Flood-Page and colleagues (57) showed a trend towards reducing severe asthma exacerbations with the higher dose of mepolizumab. However, it should be noted that the study was not sufficiently powered to show a difference in exacerbations. Management strategies that aim at reducing eosinophilic airway inflammation have been associated with a reduction in the frequency of exacerbations (59, 60). Accordingly, these studies suggest that in a subgroup of patients, eosinophils play an important role to the pathophysiology of asthmatic exacerbations. These patients are good candidates for targeted IL-5 therapy. Taking into consideration these two issues, two studies were designed and published in 2009. In the first study, asthmatic patients who had sputum eosinophilia and airway symptoms despite continued treatment with oral prednisone and high-dose ICS were stratified into two groups according to their daily dose of prednisolone (>10 or <10 mg), and then randomised to receive either mepolizumab (750 mg intravenously, *n*=9) or placebo (150 mL 0.9% saline, *n*=11) as five monthly infusions. After a 6-week run-in period, prednisolone dose reduction was attempted according to a predefined protocol. Mepolizumab reduced the number of blood and sputum eosinophils and allowed prednisone sparing without the development of asthma exacerbations. However, the study was small including only 20 patients and, thus, it could not be considered clinically directive (61). The second study included 61 subjects with refractory eosinophilic asthma despite maximum tolerated therapy, which in many cases included regular use of oral corticosteroids and a history of recurrent severe exacerbations. After a run-in period of 2 weeks prednisolone (1 mg/kg to a maximum of 40 mg), asthmatics were randomised to receive 12 infusions of either mepolizumab (750 mg intravenously, *n*=29) or placebo (150 mL 0.9% saline, *n*=32) at monthly intervals. Mepolizumab was associated with significantly fewer severe exacerbations and a significant improvement in the AQLQ score. This effect was accompanied by significantly lower eosinophil counts in blood and sputum. On the other hand, there were no significant differences in symptoms, lung function and airway hyperresponsiveness (62). No serious adverse events were recorded in either of the two studies. Although both studies demonstrated a statistically significant reduction in the rate of asthma exacerbations accompanied by a significant reduction in blood and sputum eosinophils, none of them identified any clinically meaningful improvement in symptoms, FEV_1_ or asthma control. The reduction of exacerbations by mepolizumab adds support to the role of eosinophils in the pathogenesis of severe asthma exacerbations in this particular asthmatic population.

A *post hoc* analysis of the study by Haldar et al. (62) showed that those asthmatics who responded well to higher dose oral prednisone tended to do better with mepolizumab treatment. On the other hand, asthmatic patients with marked bronchodilator reversibility showed a poorer response to mepolizumab. The message than one can derive from this observation is that mepolizumab works best in patients who have airflow limitation and symptoms as a result of corticosteroid-responsive airway inflammation rather than airway smooth muscle contraction (63).

### The new era for anti-IL-5

A multicentre, double-blind, placebo-controlled trial (DREAM) conducted at 81 centres in 13 countries included patients with a history of recurrent severe asthma exacerbations and signs of eosinophilic inflammation. All patients received 13 infusions of mepolizumab at 4-week intervals, and the primary outcome was the rate of clinically significant asthma exacerbations, defined as episodes of acute asthma requiring treatment with oral corticosteroids, admission, or a visit to an emergency department. Mepolizumab was effective and well tolerated in reducing the risk of asthma exacerbations. Moreover, mepolizumab lowered blood and sputum eosinophil counts. However, a small effect on traditional markers of asthma control such as FEV_1_, AQLQ and asthma control questionnaire (ACQ) scores was noted. This could be partly explained by the fact that measures of asthma control or quality of life are not associated with improvements elicited by reduced eosinophilic airway inflammation. Accordingly, a dissociation between symptoms and risk of exacerbations is probably evident in some patients with severe asthma. This study also provided an important clue regarding the efficacy of mepolizumab; a multivariate analysis identified that baseline peripheral blood eosinophil count and exacerbation frequency in the previous year were associated with efficacy while more traditional markers of asthma such as FEV_1_ and the acute bronchodilator response were not. Moreover, IgE concentrations and atopic status at baseline were not associated with a response to mepolizumab, potentially addressing differences in the mechanism of action and not excluding complementary effect if omalizumab and mepolizumab were used in combination (64). In a *post hoc* analyse including patients from the MENSA and the SIRIUS study, it was shown that the response to mepolizumab in reducing the rate of exacerbations was the same in those who had or had not been previously treated with omalizumab (65). Moreover, in the SIRIUS study, the reductions in the use of oral corticosteroids (OCS) were comparable regardless of prior omalizumab use (65). Importantly, adverse events were also comparable irrespective of prior omalizumab use (65).

Using a specific hematologic and phenotyping approach, according to the findings of the DREAM study, asthmatic patients with recurrent asthma exacerbations and evidence of eosinophilic inflammation, treated with high-dose ICS with or without maintenance oral glucocorticoids, were selected to receive mepolizumab as either a 75-mg intravenous dose or a 100-mg subcutaneous dose, or placebo every 4 weeks for 32 weeks. The MENSA study was a multicentre, randomised, double-blind, double-dummy, phase 3, placebo-controlled trial involving 576 patients. The primary outcome was the rate of exacerbations. Mepolizumab administered either intravenously or subcutaneously significantly reduced asthma exacerbations by approximately one half and was associated with improvements in quality of life and asthma control. Both intravenous and subcutaneous doses were effective and had acceptable side effect profiles (66).

Another study by Bel et al. included asthmatics with a higher rate of daily oral glucocorticoid use (100% vs. 25% of the study group in the MENSA study). Many severe asthmatics require regular treatment with oral corticosteroids resulting in serious and often irreversible adverse effects. In this study, called the Steroid Reduction with Mepolizumab Study (SIRIUS), the effect of mepolizumab adjunctive subcutaneous therapy in reducing the use of maintenance oral corticosteroids while maintaining asthma control in 135 patients with severe eosinophilic asthma was examined (67). An advantage of this study was that an optimisation phase was incorporated for the patients’ oral corticosteroid regimen. In this way, doses of oral corticosteroids were reduced as much as possible before starting mepolizumab treatment, thus providing the assurance that asthmatics genuinely required oral corticosteroids for control of their asthma. The primary outcome was the percentage reduction in the corticosteroid dose (90 to 100% reduction, 75 to less than 90% reduction, 50 to less than 75% reduction, more than 0 to less than 50% reduction, no decrease in oral corticosteroid dose, a lack of asthma control during Weeks 20–24 or withdrawal from treatment). Mepolizumab treatment led to significantly greater reductions in the maintenance oral corticosteroid dose than placebo. Moreover, despite receiving a reduced corticosteroid dose, patients in the mepolizumab group, as compared with those in the placebo group, had a relative reduction of 32% in the annualised rate of exacerbations. Mepolizumab also had a significantly beneficial effect on asthma control, and quality of life, even though patients had a clinically relevant reduction in the dose of oral corticosteroids.

An unblinded, prospective, observational study was performed as part of a follow-up, including subjects who had completed a 12-month administration of mepolizumab in refractory asthma to evaluate the kinetics of blood and sputum eosinophil counts and assess the possible relationship between such changes and the clinical course of the disease. These subjects were observed for 12 months with assessments every 3 months. Cessation of mepolizumab was associated with a rise in the blood eosinophil count soon after stopping therapy and continuing to baseline over 6 months. The frequency of severe exacerbations also increased significantly after stopping mepolizumab. The rise in exacerbations at 3–6 months after stopping mepolizumab was preceded by a rise in sputum and blood eosinophils, supporting that these events have different time courses (68).

## Discussion

The options provided for treatment Step 5 (GINA) are anti-IgE and oral corticosteroids. The latter are associated with many and sometimes detrimental adverse effects, and the lower possible dose is recommended (ideally<7.5 mg prednisolone/day) (1). Anti-IgE (omalizumab) is the recommended treatment for allergic asthma, and this treatment has been associated with reduction of exacerbations and improvement of quality of life (26). New treatments are being tested and are becoming available for severe asthmatics. The most recent one is anti-IL-5 which was found to be particularly effective in severe eosinophilic asthma.

New treatments for severe asthma are mainly monoclonal antibodies and constitute an expensive treatment choice. However, their indication applies only to a minority of asthmatics, those with severe refractory to treatment disease. These patients comprise the majority of asthma cost. If the adverse effects of systemic corticosteroids are taken under consideration, then such treatments may prove cost-effective mainly because of the significant reduction of exacerbations leading to fewer hospital admissions, emergency visits and unscheduled doctor visits (69).

A reasonable question is whether in a newly encountered case of severe asthma anti-IgE or anti-IL-5 should be the first choice. Currently, no studies have been performed to compare the effect difference of these two antibodies on severe asthma. It is conceivable that in non-allergic eosinophilic asthma, there is no place for anti-IgE treatment although occasionally omalizumab has been administered in non-allergic asthmatics based on local production of IgE (70, 71).

However, in case of allergic eosinophilic asthma, what might be the first treatment? Mepolizumab almost depletes eosinophils from peripheral blood and significantly reduces them from the airways, and omalizumab also reduces sputum and tissue eosinophils, as it has been shown in lung biopsy studies (14, 58). In a pooled analysis from five randomised, double-blind, placebo-controlled studies including patients with moderate-to-severe persistent allergic asthma, omalizumab was associated with significantly reduced post-treatment peripheral blood eosinophil counts (13). Moreover, the greater reductions were observed in those patients with lower post-treatment free IgE levels. In the EXTRA study including 850 patients evaluating the peripheral blood eosinophil count as predictor of treatment effect of omalizumab, it was found that omalizumab was more effective in reducing exacerbation frequency in the high (>260 cells/µL) blood eosinophil group compared with that in the low blood eosinophil group (43). In the same study, high FeNO and high periostin were also predictors of response to omalizumab treatment.

It is likely that some patients with severe asthma have been treated with anti-IgE, and omalizumab was stopped after 16 weeks because of an unfavourable effect. In that case, mepolizumab may be the choice.

In the larger study regarding mepolizumab, the DREAM study, it was demonstrated that only two variables were associated with efficacy, and these were baseline peripheral blood eosinophil count and exacerbation frequency in the previous year. The higher these were, the more effective the treatment was (64). Accordingly, in a case with very high blood eosinophil counts, mepolizumab may be the first choice.

IgE has been shown to increase airway remodelling in asthma through increased airway smooth muscle proliferation and deposition of proinflammatory collagens and fibronectin. Recent studies have shown that long treatment with anti-IgE significantly reduced airway wall thickness and RBM thickness within 6 and 12 months, and this effect was independent of eosinophilic infiltration (21, 22). Moreover, in another study, it was demonstrated that 48 weeks of treatment with omalizumab resulted in decrease in airway wall thickness as assessed by computed tomography (72). Accordingly, in a severe asthmatic with persistent airway obstruction possibly associated with airway remodelling, omalizumab may be the first choice. However, it should be stated that in a study examining the effect of anti-IL-5 in bronchial biopsies from 24 atopic asthmatics, it was demonstrated that apart from the reduction in the numbers and the percentage of airway eosinophils expressing mRNA for TGF-β1 (which has been implicated in asthma remodelling), anti-IL-5 was associated with reduction in the expression of tenascin, lumican and procollagen III in the bronchial mucosa RBM as well as with reduction of TGF-β1 concentration in bronchoalveolar lavage (BAL) (73). These findings indicate that the selective reduction of eosinophils from the airways may have a reversing effect on the remodelling process.

On the other hand, one might postulate that a combination of the two monoclonal antibodies may have a significantly stronger effect on the control of asthma in some severe asthmatics. We definitely need studies to prove it, but in the DREAM study, IgE concentrations and atopic status at baseline were not associated with the response to mepolizumab, thus potentially differentiating this treatment from omalizumab (64).

Moreover, in the study by Magnan et al. (65), it was demonstrated that patients with severe eosinophilic asthma who had previously received omalizumab responded positively to mepolizumab.

The future is linked with the need for direct comparisons of anti-IgE and anti-IL-5 against another along with the search for new biomarkers that will have a better ability to predict response to treatment either alone or in combination with the existing ones.

## References

[1] Reddel HK, Bateman ED, Becker A, Boulet LP, Cruz AA, Drazen JM (2015). A summary of the new GINA strategy: a roadmap to asthma control. Eur Respir J.

[2] Chung KF, Wenzel SE, Brozek JL, Bush A, Castro M, Sterk PJ (2014). International ERS/ATS guidelines on definition, evaluation and treatment of severe asthma. Eur Respir J.

[3] Kane B, Fowler SJ, Niven R (2015). Refractory asthma – beyond step 5, the role of new and emerging adjuvant therapies. Chron Respir Dis.

[4] Menzella F, Lusuardi M, Galeone C, Zucchi L (2015). Tailored therapy for severe asthma. Multidiscip Respir Med.

[5] Rubinsztajn R, Chazan R (2016). Monoclonal antibodies for the management of severe asthma. Adv Exp Med Biol.

[6] Presta LG, Lahr SJ, Shields RL, Porter JP, Gorman CM, Fendly BM (1993). Humanization of an antibody directed against IgE. J Immunol.

[7] Chang TW (2006). Developing antibodies for targeting immunoglobulin and membrane-bound immunoglobulin E. Allergy Asthma Proc.

[8] Chang TW, Shiung YY (2006). Anti-IgE as a mast cell-stabilizing therapeutic agent. J Allergy Clin Immunol.

[9] Chang TW (2000). The pharmacological basis of anti-IgE therapy. Nat Biotechnol.

[10] 
Laffer S, Lupinek C, Rauter I, Kneidinger M, Drescher A, Jordan JH (2008). A high-affinity monoclonal anti-IgE antibody for depletion of IgE and IgE-bearing cells. Allergy.

[11] MacGlashan DW, Bochner BS, Adelman DC, Jardieu PM, Togias A, McKenzie-White J (1997). Down-regulation of Fc(epsilon) RI expression on human basophils during *in vivo* treatment of atopic patients with anti-IgE antibody. J Immunol.

[12] Prussin C, Griffith DT, Boesel KM, Lin H, Foster B, Casale TB (2003). Omalizumab treatment downregulates dendritic cell Fcepsilon-RI expression. J Allergy Clin Immunol.

[13] Massanari M, Holgate ST, Busse WW, Jimenez P, Kianifard F, Zeldin R (2010). Effect of omalizumab on peripheral blood eosinophilia in allergic asthma. Respir Med.

[14] Djukanovic R, Wilson SJ, Kraft M, Jarjour NN, Steel M, Chung KF (2004). Effects of treatment with anti-immunoglobulin E antibody omalizumab on airway inflammation in allergic asthma. Am J Respir Crit Care Med.

[15] Yalcin AD, Cilli A, Bisgin A, Strauss LG, Herth F (2013). Omalizumab is effective in treating severe asthma in patients with severe cardiovascular complications and its effects on sCD200, d-dimer, CXCL8, 25-hydroxyvitamin D and IL-1beta levels. Expert Opin Biol Ther.

[16] Yalcin AD (2014). An overview of the effects of anti-IgE therapies. Med Sci Monit.

[17] Silkoff PE, Romero FA, Gupta N, Townley RG, Milgrom H (2004). Exhaled nitric oxide in children with asthma receiving Xolair (omalizumab), a monoclonal anti-immunoglobulin E antibody. Pediatrics.

[18] Zietkowski Z, Skiepko R, Tomasiak-Lozowska MM, Bodzenta-Lukaszyk A (2010). Anti-IgE therapy with omalizumab decreases endothelin-1 in exhaled breath condensate of patients with severe persistent allergic asthma. Respiration.

[19] Xu J, Zhong NS (1999). Mechanisms of bronchial hyperresponsiveness: the interaction of endothelin-1 and other cytokines. Respirology.

[20] Kang JY, Kim JW, Kim JS, Kim SJ, Lee SH, Kwon SS (2010). Inhibitory effects of anti-immunoglobulin E antibodies on airway remodeling in a murine model of chronic asthma. J Asthma.

[21] Hoshino M, Ohtawa J (2012). Effects of adding omalizumab, an anti-immunoglobulin E antibody, on airway wall thickening in asthma. Respiration.

[22] Riccio AM, Dal Negro RW, Micheletto C, De Ferrari L, Folli C, Chiappori A (2012). Omalizumab modulates bronchial reticular basement membrane thickness and eosinophil infiltration in severe persistent allergic asthma patients. Int J Immunopathol Pharmacol.

[23] Mauri P, Riccio AM, Rossi R, Di Silvestre D, Benazzi L, De Ferrari L (2014). Proteomics of bronchial biopsies: galectin-3 as a predictive biomarker of airway remodelling modulation in omalizumab-treated severe asthma patients. Immunol Lett.

[24] Bousquet J, Cabrera P, Berkman N, Buhl R, Holgate S, Wenzel S (2005). The effect of treatment with omalizumab, an anti-IgE antibody, in asthma exacerbations and emergency medical visits in patients with severe persistent asthma. Allergy.

[25] Humbert M, Beasley R, Ayres J, Slavin R, Hebert J, Bousquet J (2005). Benefits of omalizumab as add-on therapy in patients with severe persistent asthma who are inadequately controlled despite best available therapy (GINA 2002 step 4 treatment): INNOVATE. Allergy.

[26] Normansell R, Walker S, Milan SJ, Walters EH, Nair P (2014). Omalizumab for asthma in adults and children. Cochrane Database Syst Rev.

[27] Abraham I, Alhossan A, Lee CS, Kutbi H, MacDonald K (2016). ‘Real-life’ effectiveness studies of omalizumab in adult patients with severe allergic asthma: systematic review. Allergy.

[28] Busse WW, Morgan WJ, Gergen PJ, Mitchell HE, Gern JE, Liu AH (2011). Randomized trial of omalizumab (anti-IgE) for asthma in inner-city children. N Engl J Med.

[29] Hanania NA, Alpan O, Hamilos DL, Condemi JJ, Reyes-Rivera I, Zhu J (2011). Omalizumab in severe allergic asthma inadequately controlled with standard therapy: a randomized trial. Ann Intern Med.

[30] Chen H, Eisner MD, Haselkorn T, Trzaskoma B (2013). Concomitant asthma medications in moderate-to-severe allergic asthma treated with omalizumab. Respir Med.

[31] Tzortzaki EG, Georgiou A, Kampas D, Lemessios M, Markatos M, Adamidi T (2012). Long-term omalizumab treatment in severe allergic asthma: the South-Eastern Mediterranean ‘real-life’ experience. Pulm Pharmacol Ther.

[32] Vennera Mdel C, Perez De Llano L, Bardagi S, Ausin P, Sanjuas C, Gonzalez H (2012). Omalizumab therapy in severe asthma: experience from the Spanish registry – some new approaches. J Asthma.

[33] Corren J, Casale TB, Lanier B, Buhl R, Holgate S, Jimenez P (2009). Safety and tolerability of omalizumab. Clin Exp Allergy.

[34] Rodrigo GJ, Neffen H, Castro-Rodriguez JA (2011). Efficacy and safety of subcutaneous omalizumab vs. placebo as add-on therapy to corticosteroids for children and adults with asthma: a systematic review. Chest.

[35] Tan RA, Corren J (2011). Safety of omalizumab in asthma. Expert Opin Drug Saf.

[36] Busse W, Buhl R, Fernandez Vidaurre C, Blogg M, Zhu J, Eisner MD (2012). Omalizumab and the risk of malignancy: results from a pooled analysis. J Allergy Clin Immunol.

[37] Long A, Rahmaoui A, Rothman KJ, Guinan E, Eisner M, Bradley MS (2014). Incidence of malignancy in patients with moderate-to-severe asthma treated with or without omalizumab. J Allergy Clin Immunol.

[38] Long AA, Fish JE, Rahmaoui A, Miller MK, Bradley MS, Taki HN (2009). Baseline characteristics of patients enrolled in EXCELS: a cohort study. Ann Allergy Asthma Immunol.

[39] Ali AK, Hartzema AG (2012). Assessing the association between omalizumab and arteriothrombotic events through spontaneous adverse event reporting. J Asthma Allergy.

[40] Cruz AA, Lima F, Sarinho E, Ayre G, Martin C, Fox H (2007). Safety of anti-immunoglobulin E therapy with omalizumab in allergic patients at risk of geohelminth infection. Clin Exp Allergy.

[41] Bousquet J, Wenzel S, Holgate S, Lumry W, Freeman P, Fox H (2004). Predicting response to omalizumab, an anti-IgE antibody, in patients with allergic asthma. Chest.

[42] Bousquet J, Rabe K, Humbert M, Chung KF, Berger W, Fox H (2007). Predicting and evaluating response to omalizumab in patients with severe allergic asthma. Respir Med.

[43] Hanania NA, Wenzel S, Rosen K, Hsieh HJ, Mosesova S, Choy DF (2013). Exploring the effects of omalizumab in allergic asthma: an analysis of biomarkers in the EXTRA study. Am J Respir Crit Care Med.

[44] 
Nopp A, Johansson SG, Ankerst J, Palmqvist M, Oman H (2007). CD-sens and clinical changes during withdrawal of Xolair after 6 years of treatment. Allergy.

[45] Nopp A, Johansson SG, Adedoyin J, Ankerst J, Palmqvist M, Oman H (2010). After 6 years with Xolair; a 3-year withdrawal follow-up. Allergy.

[46] Bousquet J, Chanez P, Lacoste JY, Barneon G, Ghavanian N, Enander I (1990). Eosinophilic inflammation in asthma. N Engl J Med.

[47] Bousquet J, Jeffery PK, Busse WW, Johnson M, Vignola AM (2000). Asthma. From bronchoconstriction to airways inflammation and remodeling. Am J Respir Crit Care Med.

[48] Wenzel SE, Schwartz LB, Langmack EL, Halliday JL, Trudeau JB, Gibbs RL (1999). Evidence that severe asthma can be divided pathologically into two inflammatory subtypes with distinct physiologic and clinical characteristics. Am J Respir Crit Care Med.

[49] Sanderson CJ (1992). Interleukin-5, eosinophils, and disease. Blood.

[50] Vancheri C, Gauldie J, Bienenstock J, Cox G, Scicchitano R, Stanisz A (1989). Human lung fibroblast-derived granulocyte-macrophage colony stimulating factor (GM-CSF) mediates eosinophil survival *in vitro*. Am J Respir Cell Mol Biol.

[51] Farahi N, Cowburn AS, Upton PD, Deighton J, Sobolewski A, Gherardi E (2007). Eotaxin-1/CC chemokine ligand 11: a novel eosinophil survival factor secreted by human pulmonary artery endothelial cells. J Immunol.

[52] Garcia G, Taille C, Laveneziana P, Bourdin A, Chanez P, Humbert M (2013). Anti-interleukin-5 therapy in severe asthma. Eur Respir Rev.

[53] Liu AH, Anderson WC, Dutmer CM, Searing DA, Szefler SJ (2016). Advances in asthma 2015: across the lifespan. J Allergy Clin Immunol.

[54] Leckie MJ, ten Brinke A, Khan J, Diamant Z, O'Connor BJ, Walls CM (2000). Effects of an interleukin-5 blocking monoclonal antibody on eosinophils, airway hyper-responsiveness, and the late asthmatic response. Lancet.

[55] Kips JC, O'Connor BJ, Langley SJ, Woodcock A, Kerstjens HA, Postma DS (2003). Effect of SCH55700, a humanized anti-human interleukin-5 antibody, in severe persistent asthma: a pilot study. Am J Respir Crit Care Med.

[56] O'Byrne PM, Inman MD, Parameswaran K (2001). The trials and tribulations of IL-5, eosinophils, and allergic asthma. J Allergy Clin Immunol.

[57] Flood-Page P, Swenson C, Faiferman I, Matthews J, Williams M, Brannick L (2007). A study to evaluate safety and efficacy of mepolizumab in patients with moderate persistent asthma. Am J Respir Crit Care Med.

[58] Flood-Page PT, Menzies-Gow AN, Kay AB, Robinson DS (2003). Eosinophil's role remains uncertain as anti-interleukin-5 only partially depletes numbers in asthmatic airway. Am J Respir Crit Care Med.

[59] Green RH, Brightling CE, McKenna S, Hargadon B, Parker D, Bradding P (2002). Asthma exacerbations and sputum eosinophil counts: a randomised controlled trial. Lancet.

[60] Jayaram L, Pizzichini MM, Cook RJ, Boulet LP, Lemiere C, Pizzichini E (2006). Determining asthma treatment by monitoring sputum cell counts: effect on exacerbations. Eur Respir J.

[61] Nair P, Pizzichini MM, Kjarsgaard M, Inman MD, Efthimiadis A, Pizzichini E (2009). Mepolizumab for prednisone-dependent asthma with sputum eosinophilia. N Engl J Med.

[62] Haldar P, Brightling CE, Hargadon B, Gupta S, Monteiro W, Sousa A (2009). Mepolizumab and exacerbations of refractory eosinophilic asthma. N Engl J Med.

[63] Pavord ID, Haldar P, Bradding P, Wardlaw AJ (2010). Mepolizumab in refractory eosinophilic asthma. Thorax.

[64] Pavord ID, Korn S, Howarth P, Bleecker ER, Buhl R, Keene ON (2012). Mepolizumab for severe eosinophilic asthma (DREAM): a multicentre, double-blind, placebo-controlled trial. Lancet.

[65] Magnan A, Bourdin A, Prazma CM, Albers FC, Price RG, Yancey SW (2016). Treatment response with mepolizumab in severe eosinophilic asthma patients with previous omalizumab treatment. Allergy.

[66] Ortega HG, Liu MC, Pavord ID, Brusselle GG, FitzGerald JM, Chetta A (2014). Mepolizumab treatment in patients with severe eosinophilic asthma. N Engl J Med.

[67] Bel EH, Wenzel SE, Thompson PJ, Prazma CM, Keene ON, Yancey SW (2014). Oral glucocorticoid-sparing effect of mepolizumab in eosinophilic asthma. N Engl J Med.

[68] Haldar P, Brightling CE, Singapuri A, Hargadon B, Gupta S, Monteiro W (2014). Outcomes after cessation of mepolizumab therapy in severe eosinophilic asthma: a 12-month follow-up analysis. J Allergy Clin Immunol.

[69] Braunstahl GJ, Chen CW, Maykut R, Georgiou P, Peachey G, Bruce J (2013). The eXpeRience registry: the ‘real-world’ effectiveness of omalizumab in allergic asthma. Respir Med.

[70] Garcia G, Magnan A, Chiron R, Contin-Bordes C, Berger P, Taille C (2013). A proof-of-concept, randomized, controlled trial of omalizumab in patients with severe, difficult-to-control, nonatopic asthma. Chest.

[71] de Llano LP, Vennera Mdel C, Alvarez FJ, Medina JF, Borderias L, Pellicer C (2013). Effects of omalizumab in non-atopic asthma: results from a Spanish multicenter registry. J Asthma.

[72] Tajiri T, Niimi A, Matsumoto H, Ito I, Oguma T, Otsuka K (2014). Comprehensive efficacy of omalizumab for severe refractory asthma: a time-series observational study. Ann Allergy Asthma Immunol.

[73] Flood-Page P, Menzies-Gow A, Phipps S, Ying S, Wangoo A, Ludwig MS (2003). Anti-IL-5 treatment reduces deposition of ECM proteins in the bronchial subepithelial basement membrane of mild atopic asthmatics. J Clin Invest.

